# Progressive multifocal leukoencephalopathy in rituximab-treated rheumatic diseases: a rare event

**DOI:** 10.1007/s13365-018-0615-7

**Published:** 2018-03-05

**Authors:** Joseph R. Berger, Vineeta Malik, Stuart Lacey, Paul Brunetta, Patricia B. Lehane

**Affiliations:** 1 Department of Neurology, Perelman School of Medicine, University of Pennsylvania, 3400 Convention Avenue, Room 765, Philadelphia, PA 19104 USA; 2 F. Hoffmann-La Roche, Ltd., Basel, Switzerland; 3 Roche Products Ltd., Welwyn Garden City, UK; 4 Genentech, Inc, South San Francisco, USA

**Keywords:** Rituximab, Progressive multifocal leukoencephalopathy, Rheumatoid arthritis, Granulomatosis with polyangiitis, Microscopic polyangiitis

## Abstract

This report assesses the observed risk of PML in patients treated with the anti-CD20 monoclonal antibody rituximab in the regulatory authority-approved autoimmune indications rheumatoid arthritis (RA), granulomatosis with polyangiitis (GPA), and microscopic polyangiitis (MPA). This was a cumulative analysis of confirmed PML cases in patients receiving rituximab for RA or GPA/MPA from both spontaneous reports and clinical trial sources, as captured in the manufacturer global company safety and clinical databases. Overall reporting rates were calculated and patient case details were summarized. As of 17 November 2015, there were nine confirmed PML cases among patients who had received rituximab for RA and two for GPA. Corresponding estimated reporting rates were 2.56 per 100,000 patients with RA (estimated exposure ≈ 351,396 patients) and < 1 per 10,000 patients with GPA/MPA (estimated exposure 40,000–50,000 patients). In all cases, patients had ≥ 1 potential risk factor for PML independent of rituximab treatment. In the RA population, the estimated reporting rate of PML generally remained stable and low since 2009 despite increasing rituximab exposure. There was no pattern of latency from time of rituximab initiation to PML development and no association of PML with the number of rituximab courses. Global post-marketing safety and clinical trial data demonstrated that the occurrence of PML is very rare among rituximab-treated patients with RA or GPA/MPA and has remained stable over time.

## Introduction

Treatment with immunosuppressive biological agents has been reported as a potential risk factor for progressive multifocal leukoencephalopathy (PML), a rare, often fatal demyelinating disease of the central nervous system caused by the John Cunningham virus.

**Fig. 1:**
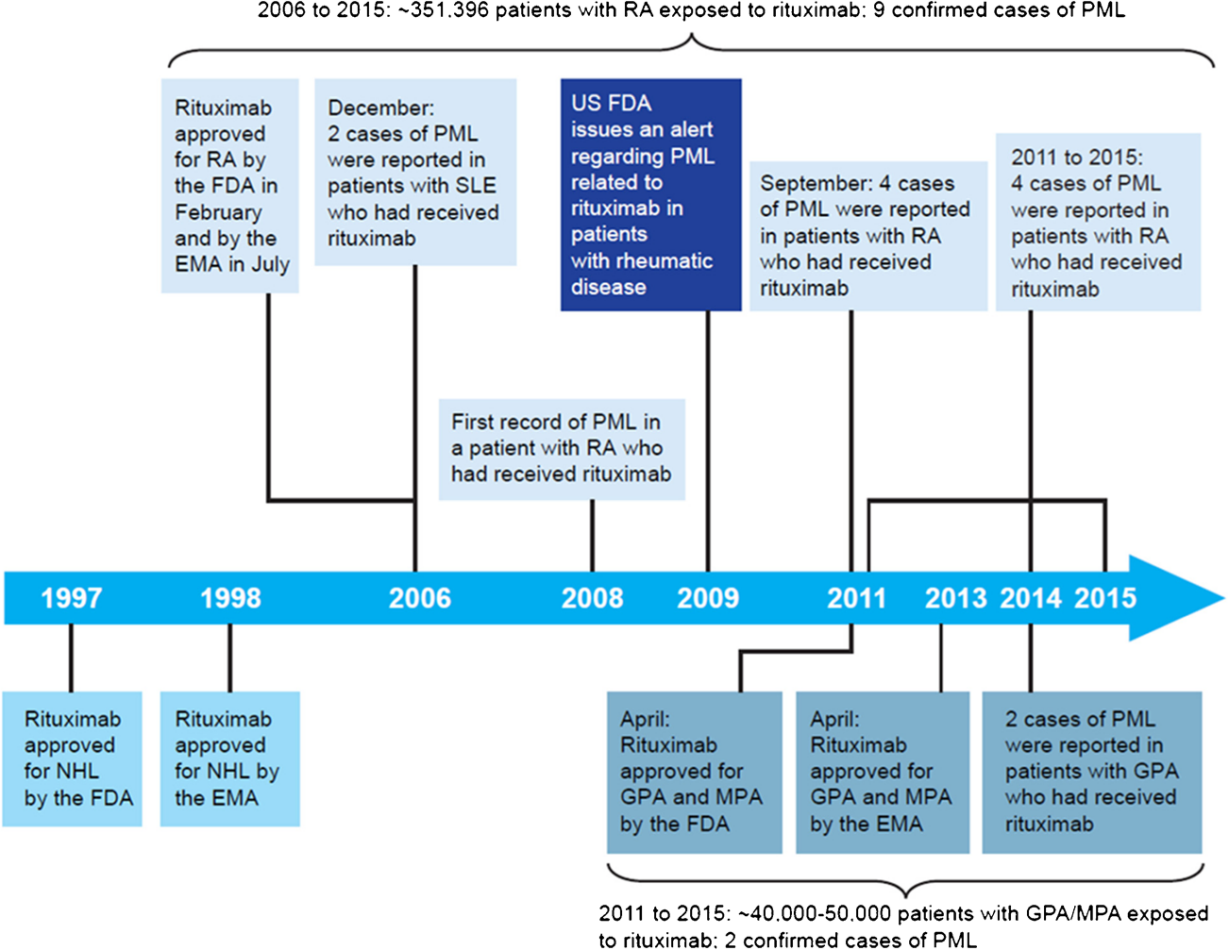
History of rituximab and PML in patients with RA or GPA/MPA. References: 2006 SLE cases (Rituxan warning 2007); 2009 RA case (Fleischmann 2009); 2011 RA cases (Clifford et al. 2011); US FDA alert. SLE is not an approved indication for rituximab; two initial cases prompted an FDA warning on PML risk following rituximab treatment. *EMA,* European Medicines Agency, *FDA,* Food and Drug Administration, *GPA*, granulomatosis with polyangiitis, *MPA,* microscopic polyangiitis, *NHL,* non-Hodgkin lymphoma, *PML,* progressive multifocal leukoencephalopathy, *RA,* rheumatoid arthritis, *SLE,* systemic lupus erythematosus

Rituximab is a chimeric anti-CD20 monoclonal antibody that targets and depletes CD20+ B cells. Initially approved worldwide to treat non-Hodgkin lymphoma and chronic lymphocytic leukemia, rituximab was subsequently approved in combination with methotrexate (MTX) for the treatment of adult patients with moderate to severely active rheumatoid arthritis (RA) who have had an inadequate response to ≥ 1 tumor necrosis factor inhibitor therapies. Rituximab later achieved global regulatory approval in combination with glucocorticoids for induction of remission in the anti-neutrophil cytoplasmic autoantibody (ANCA)-associated vasculitides: granulomatosis with polyangiitis (GPA) and microscopic polyangiitis (MPA) (Fig. 1) (Rituxan PI  2014, MabThera PI 2016). Although PML in patients receiving rituximab is well-described in the oncology setting (non-Hodgkin lymphoma or chronic lymphocytic leukemia) (Carson et al. 2009), these diseases are themselves well-known PML risk factors. The case reports of patients with autoimmune disorders who developed PML after exposure to rituximab suggested a potential association with rituximab; (2007; Clifford et al. 2011; Fleischmann 2009; Molloy and Calabrese 2012) however, these patients all had confounding factors, including predisposing comorbidities and past and concomitant therapies.

To better describe the apparent association between rituximab and PML in the autoimmune setting, this report presents a summary of confirmed PML cases and corresponding crude reporting rates among patients who received rituximab for the regulatory authority-approved autoimmune indications RA and GPA/MPA, as captured from both the manufacturer global clinical trial and safety databases (including post-marketing spontaneous reports).

## Patients and methods

### Overall strategy

A cumulative analysis of reported cases of confirmed PML in patients receiving rituximab for RA or GPA/MPA, drawing from both spontaneous reports and clinical trial sources from the manufacturer global company safety and clinical databases was undertaken.

A confirmed PML diagnosis was defined according to clinical features, characteristic changes on magnetic resonance imaging or computed tomography scans, and confirmation of the presence of JCV as detected by in situ hybridization from brain tissue biopsy, by polymerase chain reaction in CSF, or in postmortem autopsy findings.

### Analysis of the rituximab (MabThera®/Rituxan®) global safety database

This global safety database includes serious adverse events documented in the rituximab clinical trial programs for RA and GPA/MPA, as well as all spontaneously reported adverse events for which rituximab was considered a suspect drug. A search of PML events and PML confirmatory tests was carried out using MedDRA Version 18.1. The search period was from 2002 (to cover first exposure to rituximab in RA clinical trials) until the data cutoff, 17 November 2015. Potential cases were medically reviewed, and only confirmed PML cases (as defined in “Overall Strategy”) were included in this analysis. Only events occurring in the autoimmune indications approved for rituximab (RA and GPA/MPA) were included, as the overall exposure, as numbers of patients treated, could be more accurately tracked and estimated based on prescriptions for these approved indications.

### Rituximab (MabThera®/Rituxan®) clinical trial program for RA and GPA/MPA

The RA clinical trial database includes safety data from patients with moderate to severe, active RA who were treated with rituximab plus MTX within a global clinical trial program (eight randomized clinical trials, two long-term open-label extensions, and one open-label prospective study were included) (Emery et al. 2010; Edwards et al. 2004; Emery et al. 2006; Cohen et al. 2006; Keystone et al. 2007; Rubbert-Roth et al. 2010; Mease et al. 2010; Tak et al. 2011; Bingham 3rd et al. 2010; van Vollenhoven et al. 2013). Altogether, 3595 patients had received a mean of four courses (range 1–20) of rituximab over an observation period of 11 years (14,816 patient-years’ exposure) (van Vollenhoven et al. 2015). Eligibility criteria, study designs, and treatment regimens for these trials have been previously published (Emery et al. 2010; Edwards et al. 2004; Emery et al. 2006; Cohen et al. 2006; Keystone et al. 2007; Rubbert-Roth et al. 2010; Mease et al. 2010; Tak et al. 2011; Bingham 3rd et al. 2010; van Vollenhoven et al. 2013). The GPA/MPA clinical trial database included 97 patients with GPA/MPA receiving concomitant glucocorticoids in the pivotal trial “Rituximab in ANCA-Associated Vasculitis” (RAVE), for which eligibility criteria, study design, and treatment regimen have been described previously (Stone et al. 2010). The reporting window covers the primary RAVE trial period and ≥ 18 months of follow-up, up to a maximum of 5 years (Stone et al. 2010). Each database was searched for reports of confirmed PML cases. All studies were previously reported and were in compliance with the Helsinki Declaration; local institutional review board approval was given at each participating site.

### Statistical analysis

Due to the small number of PML cases, no statistical analysis was performed. Cumulative reporting rates of PML were calculated (number of confirmed PML cases per estimated patient-market exposure), and details of individual case reports were summarized.

## Results

### Reported cases of confirmed PML in patients with RA or GPA/MPA treated with rituximab

**Fig. 2:**
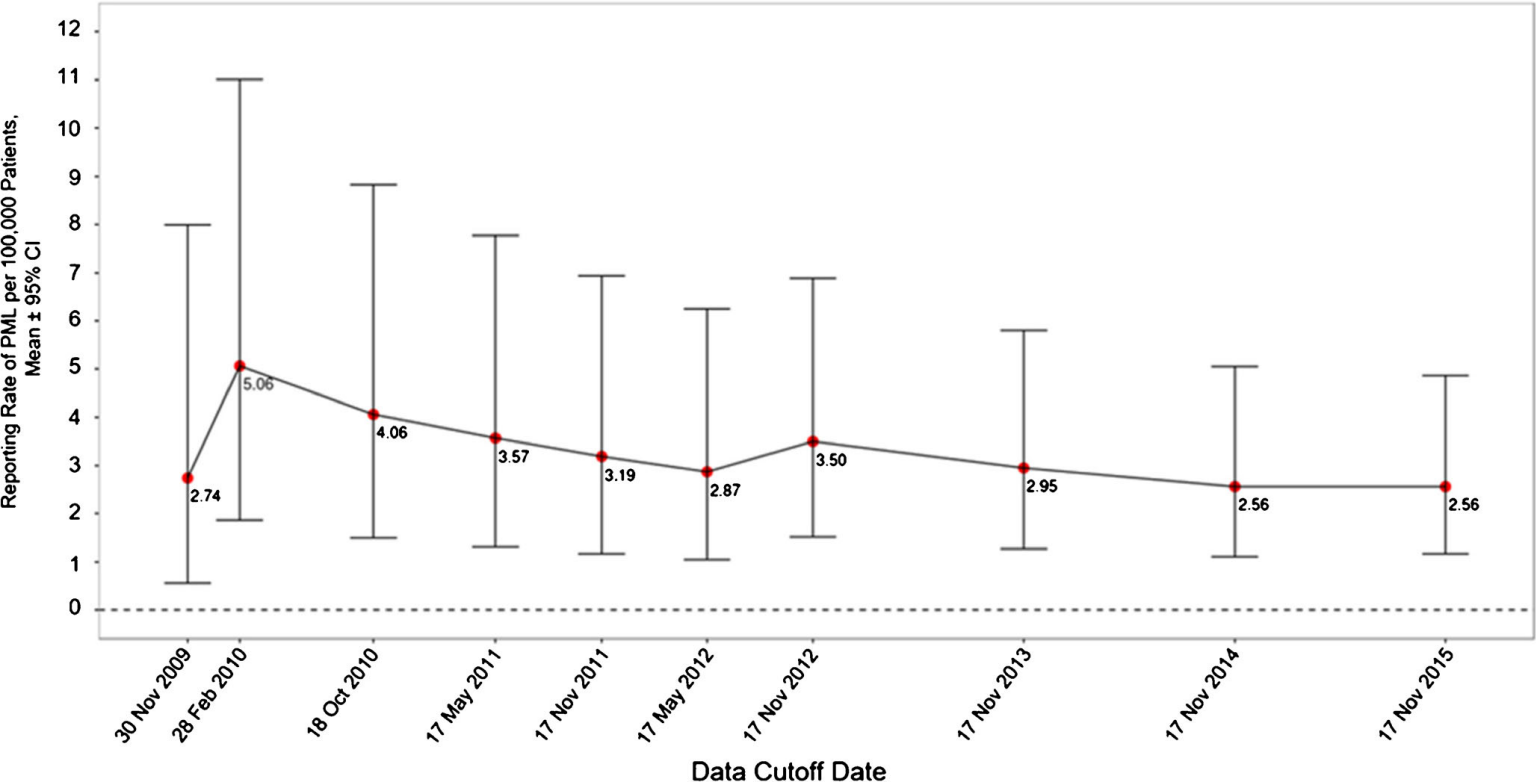
Reporting rates over time of confirmed PML cases per 100,000 patients with RA who received rituximab. Reporting rates of PML are based on estimated unique patient exposure. PML progressive multifocal leukoencephalopathy, RA rheumatoid arthritis

From the first rituximab exposure in RA clinical trials in 2002 up to a cutoff date of 17 November 2015, the company global safety database contained 11 confirmed PML cases in autoimmune disorders approved for rituximab treatment (9 in RA and 2 in GPA/MPA; Fig. 1). The 9 confirmed PML cases in patients with RA correspond to an estimated reporting rate of 2.56 cases per 100,000 patients, based on an estimated exposure of ≈ 351,396 unique patients treated with rituximab between 2006 (first approval in RA) and 2015. The change in this reporting rate over time is captured in Fig. 2. A peak in reporting rate was observed in 2010, due to most of the PML events to date being reported from 2008 to 2010 (6 out of 9; see Table 1); there has been a trend of decreasing estimated reporting rates since then, stabilizing around their current level in recent years. One of the nine reported cases was from the RA clinical trial program, previously published in 2009 (case recorded in 2008; Table 1, case 1) and occurred 5 years after the first rituximab dose and 18 months since the last dose, in a patient with a history of malignancy (Fleischmann 2009). No cases of PML occurred in the double-blind treatment period or control arms of the RA clinical trial program. Eight cases were from spontaneous post-marketing reports (2007; Roche 2015). Further details of all cases are in Table 1.

**Table 1:** Cases of confirmed PML in patients with RA

Characteristic	Case 1 (Fleischmann 2009)	Case 2 (Clifford et al. 2011)	Case 3 (Clifford et al. 2011)	Case 4 (Clifford et al. 2011)	Case 5 (Clifford et al. 2011)	Case 6	Case 7	Case 8	Case 9
Age, years	50	72	72	62	71	56	58	60	83
Sex	F	F	F	F	F	F	M	F	F
Country	USA	USA	USA	Australia	Sweden	USA	Netherlands	USA	Germany
Date PML confirmed	May 2008	Nov. 2008	Sept. 2009	Oct. 2009	Nov. 2009	March 2010	Not specified	Aug. 2012	Nov. 2014
Duration of RA, years	14	30	3	20	3	6	11	5	7
Relevant medical history	Radiation, Sjögren syndrome with lymphopenia, undetectable complement and CD4, lymphadenopathy	Sjögren syndrome	Sjögren syndrome	Leukopenia	Lymphopenia at baseline, secondary Sjögren syndrome, radiation	None reported	SLE, ANA and anti-DNA antibodies positive, opportunistic infections	SLE	None reported
History of malignancy	Yes	No	No	No	Yes	No	No	No	No
Prior nonbiologics^a, b^	MTX, steroids, HCQ, etodolac	MTX, steroids	Steroids, leflunomide, HCQ	Leflunomide, sulfasalazine, gold, HCQ, steroids	MTX, steroids	Leflunomide MTX	MTX, steroids, sulfasalazine, HCQ	Azathioprine, MTX, CYC, HCQ	MTX, Steroids
Prior biologics^a^	Infliximab	Adalimumab, etanercept	None	Adalimumab, etanercept, anakinra	None	Adalimumab, etanercept	Etanercept, infliximab	None	Denosumab
Concomitant drug^a, b^	MTX, steroids	MTX, steroids	Steroids, leflunomide, HCQ	MTX	HCQ, steroids	MTX, steroids, leflunomide	MTX, steroids	Steroids, MMF	MTX, steroids
Rituximab treatment, no. of coursesb^c^	4	5	1	≈ 4	2	3	4	9	unspecified
Latency distribution (time from first rituximab infusion to PML diagnosis)	5 years from first dose and 18 months from last dose	≈ 26 months after first dose and ≈ 2 months from last dose	≈ 7 months	≈ 18 months from first dose and ≈ 3 months from last dose	≈ 15 months from first dose. Not specified relative to last dose	≈ 23 months from first dose and ≈ 6 months from last dose	≈ 28 months from first dose and ≈ 2 months from last dose	≈ 56 months from first rituximab dose and ≈ 6 months from last rituximab dose	≈ 57 months from first rituximab dose and ≈ 8 months from last rituximab dose
PML treatment	None reported	Mefloquine	Mefloquine	Mirtazapine and mefloquine	Mirtazapine and mefloquine	Plasmapheresis and mefloquine	Mirtazapine and nitrofurantoin	None reported	None reported
Outcome	Fatal	Fatal	Fatal	Recovering	Recovering	Unknown	Fatal	Fatal	Fatal

*ANA*, antinuclear antibody; *CYC*, cyclophosphamide; F, female; *HCQ*, hydroxychloroquine; M, male; *MMF*, mycophenolate mofetil; *MTX*, methotrexate; *PML*, progressive multifocal leukoencephalopathy; *RA*, rheumatoid arthritis; *SLE*, systemic lupus erythematosus

^a^MMF is a class 2 agent and adalimumab, azathioprine, CYC, etanercept, infliximab, and MTX are class 3 agents, with respect to known or possible risk for PML

^b^MMF had a PML risk signal, and azathioprine and CYC had possible PML risk signals in a disproportionality analysis of spontaneous PML reports (Chahin and Berger 2015)

^c^Per the rituximab label recommendations for RA, one treatment course consists of 2 × 1000 mg intravenous infusions separated by 2 weeks, with treatment courses repeated every 24 weeks based on clinical evaluation, but no sooner than every 16 weeks

There were two confirmed PML cases in patients with GPA, both from spontaneous post-marketing reports (Roche 2015). No cases have been reported in patients with MPA. Due to imprecision in the patient exposure estimate in this very rare indication and the low number of confirmed cases of PML reported overall in patients with GPA, a precise reporting rate could not be calculated. However, based on the estimated cumulative exposure (40,000–50,000 patients), the rate of PML in ANCA-associated vasculitis remains < 1 in 10,000 patients. There are currently no reported cases of PML in the GPA/MPA clinical trial program in patients in either the rituximab or the control arms.

### Summaries of PML cases in patients with RA or GPA/MPA treated with rituximab

Table 1 provides a summary of the confirmed PML cases in patients who received rituximab for RA. Eight of the nine cases were in women (which is representative of the general RA patient population, which typically has three times more women than men). The overall mean age of the nine patients with PML was 65 years (range, 50–83 years) compared with a mean age of 52 years (range, 18–81 years) in the RA clinical trial program of 3595 patients. Differences between the overall estimated number of patients exposed (≈ 351,396 unique patients) and the low numbers of patients with PML should be considered when interpreting these data. All nine patients with PML had a diagnosis of RA for ≥ 3 years and were HIV-negative. Additionally, they all had ≥ 1 known risk factor for PML independent of rituximab treatment. These included a history for Sjögren syndrome (*n* = 4), a history of malignancy (*n* = 2), prior and concomitant therapy with disease-modifying anti-rheumatic drugs with established risk for PML (Molloy and Calabrese 2012) (*n* = 9; the most common of which was MTX), and treatment with ≥ 2 prior TNF inhibitors (*n* = 4). Six of the nine patients died due to PML, and the outcome for one patient is unknown. The two patients who were reported at the time of the data cutoff (17 November, 2015) as recovering were treated for PML with mirtazapine and mefloquine. The number of rituximab courses ranged from 1 to 9.

Table 2 provides a summary of the confirmed PML cases in patients who received rituximab for GPA/MPA. The mean age of the 2 patients with PML was 66 years (range, 62–70 years), compared with a mean age of 56 years (range, 19–89 years) in the 97 patients in the GPA/MPA clinical trial program. Both patients with PML had GPA for 8 years and were HIV-negative. Both also had ≥ 1 known risk factor for PML independent of rituximab treatment. Patient 1 had a history of breast cancer and treatment with chemotherapy, as well as immunoglobulin deficiency. Both patients had previously received cyclophosphamide, azathioprine, and corticosteroids, and patient 1 was receiving concomitant azathioprine. Importantly, Patient 1 showed signs of PML prior to the start of rituximab treatment, including deficits in higher cognitive functions (speech, language, memory, and abstraction ability). Both patients were treated for PML with mefloquine, mirtazapine, and/or cytarabine and cidofovir, and, at the time of the data cutoff, both were reported as recovering.

**Table 2:** Cases of confirmed PML in patients with GPA

Characteristic	Case 1	Case 2
Age, years	70	62
Sex	F	M
Country	Germany	Denmark
Date PML confirmed	July 2012	Sept. 2013
Duration of GPA, years	Not specified	8
Relevant medical history	Immunoglobulin deficiency, breast cancer, diabetes mellitus, arterial hypertension, and chronic stage III renal insufficiency	None reported
Prior treatments	CYC, epirubicin, 5-FU, prednisolone, and MTX	CYC, azathioprine, and high-dose glucocorticoids
Concomitant drug	Azathioprine	None reported
Rituximab treatment	Aug. 2011–March 2012 for GPA; no. of courses not specified	2011–Mar 2013 occasionally as needed for GPA; no. of courses not specified
Latency distribution (time from first rituximab infusion to PML diagnosis)	≈ 11 months from first dose and ≈ 4 months from last dose, symptoms prior to the start of rituximab	≈ 2 years from first dose and ≈ 6 months from the last dose
PML treatment	Immune apheresis to eliminate residual rituximab, cidofovir, mefloquine, and mirtazapine	Mefloquine, mirtazapine, and cytarabine
Outcome	≈ 1 year after PML diagnosis, the patient’s condition had improved; however, she continued to experience cognitive deficits and JCV was still detected in her CSF	≈ 3 months after PML diagnosis, the patient’s condition had improved

*5-FU*, fluorouracil; *CSF*, cerebrospinal fluid; *CYC*, cyclophosphamide; *F*, female; *GPA*, granulomatosis with polyangiitis; *JCV*, John Cunningham polyomavirus; *M*, male; *MTX*, methotrexate; *PML*, progressive multifocal leukoencephalopathy

### Latency of PML after rituximab treatment and duration of rituximab treatment

No pattern of latency was detected between the first dose of rituximab and confirmed diagnosis of PML for the patients with RA (*n* = 9) or GPA (*n* = 2). There was also no association between the number of courses of rituximab and the occurrence of PML in patients with RA (range, 1–9; Table 1). For patients with GPA, the number of rituximab courses was unspecified (Table 2).

## Discussion

This report provides a comprehensive global safety and clinical database analysis of confirmed PML cases occurring in patients exposed to rituximab for the autoimmune indications of RA and GPA/MPA. The observation period spans the first exposure to rituximab in clinical trials (from 2002 for RA and 2009 for GPA/MPA) until the data cutoff date of 17 November 2015. Our findings indicate: (1) the reported occurrence of PML is very rare in both populations (2.56 cases per 100,000 patients in RA and < 1 case per 10,000 in GPA/MPA); (2) in the RA population, the estimated reporting rate of PML appears to have generally decreased since 2010 and stabilized despite increasing rituximab exposure in patients with RA receiving multiple treatment courses over time; and (3) all confirmed PML cases were associated with other risk factors, independent of rituximab treatment. Widespread use of rituximab for RA only began following its regulatory approval in 2006. The reduction and stabilization of PML cases in patients with RA after 2010 may perhaps be explained by an increased use of rituximab as opposed to other immunosuppressive therapies that more widely employed prior to its approval for these indications which may have carried a higher risk of PML.

As reported previously (2007; Clifford et al. 2011; Fleischmann 2009; Molloy and Calabrese 2012; Hashi et al. 2008), cases of PML in patients with RA treated with rituximab are associated with confounding PML risk factors, including prior and concomitant therapies, a history of malignancy, prior or concomitant SLE, and other immune disorders (leukopenia, lymphopenia). Notably, in four of the nine cases reported here, the patient also had Sjögren syndrome. It is presently not possible to separate the contribution of these factors and any contribution of rituximab to the development of PML in these cases.

Immunosenescence, the gradual deterioration of the immune system brought on by aging, has also been suggested as a contributor to PML occurrence in patients treated with immunomodulatory therapies, with patients ≥ 44 years showing a higher prevalence of “early onset” natalizumab-associated PML (defined as PML developed prior to completing 24 natalizumab courses) than patients < 44 years old (Prosperini et al. 2016). Of 9 patients developing PML with fingolimod, another disease-modifying therapy for multiple sclerosis, all but one were older than 48 years (Berger 2017). The mean age of patients reported herein who developed PML was relatively high compared both with the mean observed in the RA and GPA/MPA global clinical trial programs and with that in the general RA and GPA/MPA populations. However, the overall numbers of confirmed PML cases are low by comparison, and therefore definitive conclusions on the effect of age as a possible risk factor cannot be drawn.

While natalizumab-associated PML usually occurs after a characteristic latency period of 18 to 24 months (Chahin and Berger 2015), the number of PML cases among rituximab-treated patients with RA or GPA/MPA remains too small to assess whether any such characteristic latency period exists with rituximab for these indications. A correlation between the number of courses of rituximab and PML occurrence might be expected if rituximab were a main driver of PML in these cases. However, the number of rituximab courses was highly variable among the nine patients with RA and PML documented here (between one and nine courses) and therefore does not provide any such evidence of causality. Similarly, there was no obvious pattern in PML latency in the cumulative confirmed PML cases where the information was reported. In addition to the lack of an apparent latent period from the time of first initiation of rituximab to the development of PML, the occurrence of PML in patients whose autoimmune underlying diseases already predispose them to PML and the exceptionally low numbers of observed cases in RA and GPA/MPA, despite widespread usage of the monoclonal antibody since regulatory authority approval, further distinguish reports of PML following rituximab use from that seen with natalizumab (Chahin and Berger 2015).

Identifying the risk of PML in any individual patient receiving a given immunosuppressive biologic treatment requires an improved understanding of the barriers to the development of PML. Although evidence from patients with HIV suggests that cell-mediated immunity has a central role in controlling JCV (Koralnik et al. 2001), the role of B cells is less clear: they may act as a potential viral reservoir or contribute to the immune response controlling JCV infection (Durali et al. 2015). Rituximab robustly achieves peripheral blood B cell depletion (Leandro et al. 2006) while the risk for PML remains low, suggesting a minimal involvement of peripheral blood B cells in JCV reactivation. In addition, rituximab administration to a patient with PML neither aggravated the disease process nor prevented clearance of JCV despite a significant B cell depletion lasting 15 months, indicating that B cells are not essential for JCV clearance and recovery (Asztely et al. 2015). Future research should seek to better elucidate the factors resulting in the development of PML and how these might be affected by specific immunosuppressive therapies—including developing an improved understanding of the localities of persistent JCV, how the neurovirulent form of JCV arises during PML pathogenesis, and how JCV is regulated in white blood cells (Wollebo et al. 2015).

PML should be ruled out in any patient displaying neurological deficits, e.g., patient 1, prior to rituximab initiation. Impaired cognitive functions in patients with RA or GPA/MPA who are receiving biologics may be more than a manifestation of aging. PML should be considered as a possible diagnosis when patients treated with rituximab for autoimmune conditions present with new onset neurological manifestations, and consultation with a neurologist is advised as clinically indicated. On suspicion of PML, it is advised that further dosing of immunosuppressive therapy, including rituximab, be suspended until PML has been excluded. Upon diagnosis of confirmed PML, rituximab must be permanently discontinued and the case should be reported and followed up per standard pharmacovigilance practices (2014, 2016). Routine JCV antibody testing for virus exposure is not approved, nor warranted, for patients with RA or GPA/MPA due to the rarity of PML in these populations (Borie and Kremer 2015). To date, JCV antibody testing for PML risk reduction has been validated only with natalizumab because the observed incidence of PML is sufficiently high enough to enable estimated risk stratification in patients with multiple sclerosis (Lee et al. 2013).

### Study limitations

Only reported and confirmed cases of PML in approved autoimmune indications (RA and GPA/MPA) are included in the analysis and estimated reporting rates. The PML case reports were limited to available safety data as reported by clinicians and may not, in all cases, contain all relevant information concerning exposure to all potential confounders. For example, high-dose corticosteroids and other immunosuppressive agents are widely used in autoimmune disorders, but information on duration of concomitant therapies was generally lacking.

In addition, the data discussed herein are largely based on spontaneous reporting of PML cases over 11 years (for RA) and 5 years (for GPA/MPA), and the number of unreported cases during these time periods is unknown. Thus, this evaluation reveals the importance of physician vigilance, prompt reporting, and long-term/continued safety follow-up for an accurate prevalence estimation of very rare adverse events such as PML.

## Conclusions

The occurrence of confirmed PML in rituximab-treated patients with RA and GPA/MPA from spontaneous reporting and clinical trial sources remains very rare (defined as an incidence < 1 in 10,000) (CIOMS 1995). The risk of PML in patients with RA, as determined in this analysis based on patient-market rituximab exposure estimates, is very low, with an overall reporting rate of 2.56 confirmed PML cases per 100,000 patients. The observed reporting rate has generally declined over time and, in recent years (from 2013 onward), has remained low and stable for the yearly company analysis of reported safety data, despite increasing rituximab exposures in patients with RA and increasing numbers of treatment courses received. In patients with GPA/MPA, the observed reporting rate of PML between 2009 (first company-sponsored clinical trials in GPA/MPA) and November 2015 was also very low (< 1 case per 10,000 patients). Analysis of PML cases in the RA and GPA/MPA patient populations shows that multiple factors independent of rituximab treatment likely contributed to the development of PML. It should also be noted that rituximab is indicated for patients with RA who concomitantly receive non-biologic immunosuppressive agents, and for patients with GPA/MPA who often receive cytotoxic immunomodulatory and immunosuppressant agents and high-dose corticosteroid therapies. These are all known risk factors for PML. This analysis provides cumulative data on PML estimated reporting rates over time and important previously unpublished information to healthcare professionals prescribing rituximab for regulatory authority-approved autoimmune indications.
